# Research on Water Quality Chemical Oxygen Demand Detection Using Laser-Induced Fluorescence Image Processing

**DOI:** 10.3390/s25051404

**Published:** 2025-02-25

**Authors:** Ying Guo, Zhaoshuo Tian, Zongjie Bi, Xiaohua Che, Songlin Yin

**Affiliations:** Institute of Marine Optoelectronic Equipment, Harbin Institute of Technology at Weihai, Weihai 264209, China; guoying0121@163.com (Y.G.); bizongjie@hit.edu.cn (Z.B.); cheexiaohua@163.com (X.C.); hitslyin@163.com (S.Y.)

**Keywords:** chemical oxygen demand, low concentration, laser-induced fluorescence, image processing, RGB color features

## Abstract

Chemical Oxygen Demand (COD) serves as a crucial metric for assessing the extent of water pollution attributable to organic substances. This study introduces an innovative approach for the detection of low-concentration COD in aqueous environments through the application of Laser-Induced Fluorescence (LIF) image processing. The technique employs an image sensor to capture fluorescence image data generated by organic compounds in water when excited by ultraviolet laser radiation. Subsequently, the COD value, indicative of the concentration of organic matter in the water, is derived via image processing techniques. Utilizing this methodology, an LIF image processing COD detection system has been developed. The system is primarily composed of a CMOS image sensor, an STM32 microprocessor, a laser emission module, and a display module. In this study, the system was employed to detect mixed solutions of sodium humate and glucose at varying concentrations, resulting in the acquisition of corresponding fluorescence images. By isolating color channels and processing the image data features, variations in RGB color characteristics were analyzed. The Partial Least Squares Regression (PLSR) analysis method was utilized to develop a predictive model for COD concentration values based on the average RGB color feature values from the characteristic regions of the fluorescence images. Within the COD concentration range of 0–12 mg/L, the system demonstrated a detection relative error of less than 10%. In summary, the system designed in this research, utilizing the LIF image processing method, exhibits high sensitivity, robust stability, miniaturization, and non-contact detection capabilities for low-concentration COD measurement. It is well-suited for rapid, real-time online water quality monitoring.

## 1. Introduction

In recent years, water resource pollution has become increasingly serious, making the detection and monitoring of water quality particularly important. Chemical Oxygen Demand (COD) is a key indicator in water quality monitoring, reflecting the degree of organic pollution in water bodies [1,2]. Currently, the predominant techniques for assessing water quality in terms of COD remain chemical in nature, with the potassium dichromate method (CODCr) and the potassium permanganate method (CODMn) being the most commonly employed. These conventional chemical COD detection methods are extensively utilized owing to their high accuracy. Nevertheless, they exhibit several drawbacks, including complex procedures, prolonged measurement durations, substantial reagent consumption, and the risk of secondary pollution [3]. For instance, the potassium dichromate method necessitates the use of strong acids and hazardous potassium dichromate reagents, which not only pose health hazards to operators but also produce chromium-containing waste liquids. If not properly managed, these waste liquids can lead to secondary environmental contamination [2,4]. Compared to chemical methods, optical methods have emerged as a prominent research focus in the field of rapid online water quality detection due to their advantages of fast detection speed, pollution-free operation, and convenience [5,6,7].

Currently, the main optical methods include absorption spectroscopy, hyperspectral analysis, and fluorescence spectroscopy. The essence of absorption spectroscopy is to calculate the COD value of water by measuring the absorbance of organic matter at a specific wavelength, and it is a commonly used method for online measurement of COD. However, when the concentration of organic matter in the solution is low, the method has low measurement accuracy and low sensitivity [8]. Wang et al. [9] proposed a new method for determining the concentration of CODCr standard solutions using ultraviolet spectrophotometry, with a detection limit of 5.7 mg/L and an optimal measurement range of 5.7 to 500 mg/L. The accuracy of detection for lower concentrations is relatively low. Radzevicius et al. [10] monitored the water quality in farm packaging houses. They established a regression model between ultraviolet absorption spectra and the concentration of organic matter in water using the PLS method. The results indicated that UV at the 320 nm wavelength can serve as an indicator for monitoring suspended solids, dissolved solids, and organic matter in water. It was observed that the absorbance values for samples with low concentrations of COD were comparatively low, with a detection limit established at 125 mg/L. The hyperspectral analysis method has the advantages of high spatial resolution, high spectral resolution, and spectral integration. However, the methods are technically complex and costly and are currently primarily used for qualitative detection and analysis of water quality [11].

The detection sensitivity of fluorescence spectroscopy is typically 10 to 1000 times higher than that of absorption spectroscopy, and its detection and analysis speed is also faster. In recent years, it has gained widespread attention in the field of water quality detection [12,13]. Bridgeman et al. [14] developed an innovative LED-based fluorescence instrument designed for the rapid assessment of drinking water quality. The findings indicated that this device delivers high-sensitivity and high-accuracy real-time water quality detection, making it suitable for swift on-site evaluations. Karavanova et al. [15] investigated the Dissolved Organic Matter in the Moscow River using fluorescence spectroscopy, demonstrating the method’s sensitivity in analyzing parameters such as COD, Biological Oxygen Demand (BOD), and organic carbon. The study’s results revealed that fluorescence spectroscopy not only facilitates the evaluation of the source, molecular weight distribution, and biodegradability of Dissolved Organic Matter (DOM) but also enables real-time monitoring of DOM’s dynamic changes in aquatic environments, thereby providing reliable technical support for water quality assessment. Goffin et al. [16] proposed an environmentally friendly alternative method for measuring the COD of soluble fraction in wastewater, utilizing three-dimensional excitation and emission matrix fluorescence spectroscopy. The findings indicated that this method demonstrates high accuracy and could serve as a reliable approach for online wastewater monitoring and laboratory analysis. Zheng et al. [17] proposed a COD analysis method for water quality based on Laser-Induced Fluorescence spectra. They used a single-wavelength semiconductor laser (with a wavelength of 405 nm) as the excitation light source and collected the emitted fluorescence through a portable fiber-optic spectrometer. Principal Component Analysis (PCA) and Partial Least Squares Regression (PLSR) algorithms were used for data dimensionality reduction and model building, respectively. The results indicated that the COD prediction errors of this model for the test set are less than 20%. However, compared with laboratory standard solutions, the organic matter in surface water is more complex, and the differences in fluorescence peaks and spectral ranges of various organic substances can lead to overlapping fluorescence peaks [18]. Therefore, simple fluorescence spectroscopy analysis may have certain errors in the results.

Considering the advantages and disadvantages of the aforementioned methods, this paper seeks to introduce a novel approach tailored for the detection of low-concentration COD. The objective is to enhance the sensitivity, accuracy, and applicability of detection, thereby offering more efficient and reliable technical support for rapid online water quality monitoring.

In recent years, the rapid advancement of technologies, particularly in electronics and computer science, has led to the widespread adoption of image processing-based water quality detection systems, which are highly regarded for their accuracy, efficiency, and non-contact nature [19]. Through the analysis of water sample images, characteristic information is extracted to achieve a rapid assessment of water quality parameters. Gaiao et al. [20] first proposed a digital image-based (DIB) titration method. This method uses digital images obtained from a CCD camera to analyze changes in the RGB components of the system, enabling the determination of HCl, H_3_PO_4_, and total alkalinity in aqueous solutions. The results obtained from this method were compared with those from spectrophotometric titration, and no significant difference was observed, indicating that the DIB method has high accuracy and reliability. Hsu [21] used the near-infrared, red, and green wavelength spectra of multispectral satellite images to monitor the water quality of reservoirs in terms of three parameters, namely chlorophyll, transparency, and total phosphorus. The satellite image data were converted into on-site water quality parameters, and the water quality was evaluated based on the standards set by the Organization for Economic Cooperation and Development (OECD) and Carlson indices, providing a comprehensive assessment of the reservoir’s ecological status. Volkan et al. [22] proposed a single-image reference method using smartphones for colorimetric water quality detection, introducing a smartphone-based platform for this purpose. The platform was tested in colorimetric measurements of four different water quality samples at varying concentration levels. The results demonstrated that the platform achieved 100% accuracy in colorimetric determination with notable color differences. Bansod et al. [23] investigated the application of hyperspectral imaging for monitoring water quality in the Ganges River. The Airborne visible/infrared imaging spectrometer new generation (AVIRIS-NG) was used to obtain hyperspectral images over the Ganges River. Then, the characteristic wavelengths were screened, and machine learning algorithms were adopted to construct prediction models between spectral data and chlorophyll, turbidity, and total phosphorus concentrations. The results showed that the prediction results based on hyperspectral imaging were highly consistent with the ground truth data obtained through conventional on-site measurements. Sun et al. [24] developed a multi-parameter portable surface water quality detection system by combining photoelectric detection techniques and UV–Vis absorption spectroscopy. After colorimetric treatment of standard solutions, images were captured and processed to obtain spectra of various concentration solutions, along with their corresponding RGB and HSV spectral curves. Using a convolutional neural network, a detection model was constructed between color feature values and the concentrations of analytes. It was able to achieve rapid detection of water quality parameters, such as phosphate and nitrite, with a prediction accuracy of 89%. Peng et al. [25] proposed a water quality detection method based on machine learning and image processing. This method extracts and segments the color features (RGB values) of samples from a dataset of water sample images. By constructing a decision tree model for the data samples and performing regression analysis to fit the color feature dataset with the ammonia nitrogen concentration in the water samples, the method establishes a correlation between the RGB color features, water turbidity, and ammonia nitrogen content. This approach enables dual-variable water quality detection with an accuracy of up to 90.24%. Currently, water quality detection systems utilizing image processing predominantly rely on absorption or reflection spectra, whereas the integration of fluorescence spectra with image processing remains relatively uncommon.

In summary, the main advantages of fluorescence analysis are high sensitivity and non-contact detection. Meanwhile, image processing not only facilitates non-contact detection but also provides a large field of view and cost-effectiveness. This study combines the advantages of fluorescence analysis and image processing to propose the fluorescence imaging method to detect the low-concentration COD parameters of water quality. In the experiment, Laser-Induced Fluorescence (LIF) was used to excite water samples, and fluorescence images were captured by an image sensor. Image processing techniques were then employed to extract and analyze features from the fluorescence images. By analyzing the RGB color features of the selected fluorescence regions, a COD concentration prediction model was constructed to achieve accurate, non-contact, and rapid online detection of low-concentration COD water quality parameters.

## 2. Detection Principle and Experimental Setup

### 2.1. Detection Principle

The LIF technique operates on the principle that samples emit fluorescence when subjected to laser excitation. Specifically, when organic molecules present in either aqueous samples or other media are exposed to ultraviolet laser radiation, the energy from the laser excites the electrons within these molecules, elevating them to an excited state. In this excited state, the molecules possess increased energy and exist in a non-equilibrium condition relative to their ground state. Subsequently, they release energy over a brief period through either non-radiative or radiative transitions, thereby returning to the ground state. This transition is accompanied by the emission of fluorescence [26].

**Figure 1:**
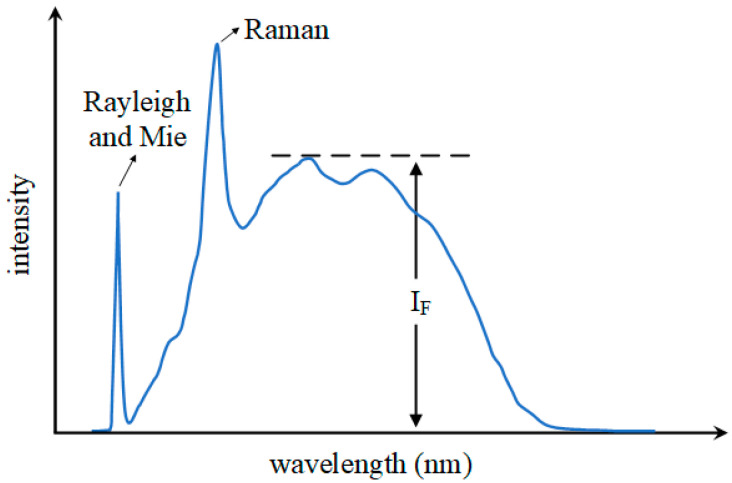
The typical spectrum produced by laser excitation of water.

The typical spectrum generated by laser incidence in water is illustrated in Figure 1. The entire spectrum primarily consists of Rayleigh scattering and Mie scattering caused by elastic scattering, water Raman scattering resulting from inelastic scattering, and the fluorescence signal *I_F_* generated by the excitation of organic matter in water [27,28].

When a laser irradiates water, the fluorescence signal typically increases with the concentration of organic matter. However, at higher concentrations, the inner filter effect may reduce the fluorescence intensity. Therefore, it is reasonable to assume that the intensity of the characteristic peak of organic matter in fluorescence is linearly correlated with the concentration of organic substances within a specific range, where the inner filter effect is negligible, as represented by the following equation:

*C* = *k* × *I_F_* + *b* (1)

In Equation (1), *C* represents the concentration of organic matter in the water sample to be measured; *I_F_* represents the spectral intensity of the characteristic peak of fluorescence; the coefficient *k* and the detection limit *b* can be determined in the experiment [29].

In numerous practical applications, imaging serves as an effective tool for visualizing the distribution and dynamics of fluorescence signals. Prior research has validated the efficacy of imaging techniques in fluorescence analysis. High-resolution imaging devices are capable of accurately capturing and recording fluorescence signals emitted by samples [30,31]. This method of data acquisition has gained widespread recognition within the field of fluorescence analysis. Following data acquisition, advanced image processing algorithms are employed to conduct in-depth analyses of the acquired fluorescence images. These algorithms facilitate a detailed and precise examination of fluorescence signal distribution across spatial and temporal dimensions [32]. By optimizing and adjusting parameters such as contrast and color gain, these algorithms can effectively extract detailed information from fluorescence signals, thereby significantly enhancing the signal-to-noise ratio and the accuracy of fluorescence signal analysis [33]. Through the synergistic application of the aforementioned imaging and image processing techniques, a correlation between fluorescence data and COD concentration has been established and substantiated in numerous studies [34,35]. This integration of technologies offers a crucial theoretical foundation and technical support for the real-time, non-contact, and rapid assessment of water quality COD utilizing Laser-Induced Fluorescence and image processing techniques [36].

### 2.2. Experimental Setup

This system integrates LIF with image processing techniques to detect organic matter in water by measuring the COD concentration. It exploits the fluorescence phenomenon that occurs when the test solution is irradiated perpendicularly by a laser.

**Figure 2:**
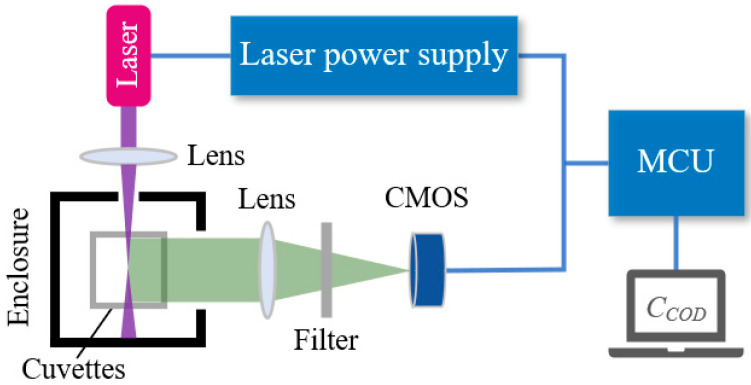
Experimental setup for COD detection.

Figure 2 illustrates the schematic diagram of the experimental setup, which is comprised of five primary components: the laser emission module, image acquisition module, data processing module, sample module, and host computer display. The laser emission module includes a 405 nm semiconductor laser, developed by Sony Corporation in Japan, with an output power ranging from 10 to 100 mW. This module also incorporates a converging lens manufactured by CHIOPT in China and a laser power supply. The laser is used to vertically irradiate the water sample, thereby inducing fluorescence. The image acquisition module employs a CMOS image sensor, model OV7725, from OmniVision Technologies in the United States, with a pixel size of 6.0 μm × 6.0 μm, to capture the fluorescence signal emitted by the water sample and obtain the fluorescence image. To achieve optimal imaging, a lens with a focal length of 12 mm, manufactured by China CHIOPT, is positioned in front of the image sensor. A long-pass filter, produced by Americanrock, is situated behind the lens to mitigate the effects of laser scattering on the fluorescence image. Within the data processing module, the Microcontroller Unit (MCU) functions as the primary data processing component. It acquires RGB image data and conducts color channel separation, feature extraction, and analytical processing on the fluorescence image data. Following this, it develops a COD concentration regression model based on the RGB color feature values derived from the fluorescence images, ultimately calculating the predicted COD value of the water sample. Furthermore, the microcontroller is tasked with controlling the laser and can adjust parameters such as exposure time, color gain, and saturation of the CMOS sensor in response to the intensity and saturation levels of the fluorescence signal. The sample module comprises a four-way optical quartz cuvette housed within a light-shielding enclosure. The cuvette is positioned within a light-shielding enclosure to minimize ambient light interference. A laser beam is directed through the cuvette containing the water sample, and fluorescence images are captured by a CMOS sensor oriented perpendicularly to the laser’s path. This enclosure effectively mitigates ambient light interference, thereby enhancing the accuracy of fluorescence signal detection by the system. Additionally, an upper computer display module is employed to present real-time measurements of COD concentrations and relevant system parameters.

**Figure 3:**
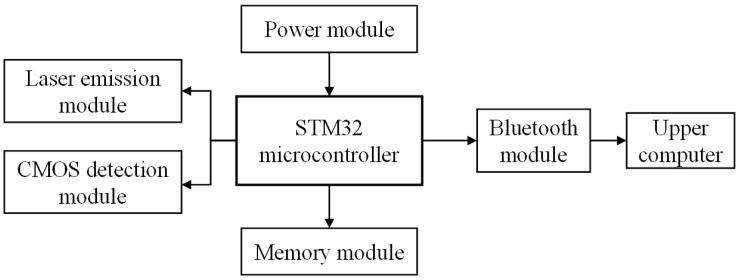
System hardware architecture diagram.

The hardware architecture of the LIF image processing COD detection instrument designed in this paper is shown in Figure 3. In this system, the STM32 microcontroller (STMicroelectronics, Geneva, Switzerland) is selected as the MCU. The laser emission module regulates the current flowing through the laser by means of a digital potentiometer, thus modulating the laser’s output power. Before performing the experiment, the output power of the laser is measured and calibrated using a power meter (VLP-2000, Beijing Yanbang Technology Co., Ltd., Beijing, China). The STM32 realizes the intensity control of the laser through the laser emission module and receives RGB images collected by the CMOS detection module through the Digital Camera Interface (DCMI) interface. The memory module is primarily used to store fluorescence images and extract regional RGB color features, ensuring real-time and rapid image and data refresh. The power module is responsible for ensuring the normal operation of the entire system. Data communication between STM32 and the upper computer is carried out via a Bluetooth module to achieve a real-time display of COD detection values.

## 3. Experimental Detection and Result Analysis

The organic matter present in natural water predominantly consists of humic substances, which are estimated to account for over 50% of the organic content [37], in addition to proteins, polysaccharides, and polypeptides. Humic-like substances exhibit significant fluorescence when excited by UV laser light. Given that their fluorescence properties are closely associated with the determination of COD in laser spectroscopy, sodium humate, as a representative of humic-like substances, serves as an appropriate substitute for the preparation of COD testing solutions in laser spectroscopy. Notably, the fluorescence intensity of sodium humate solutions is higher than that of natural water with an equivalent COD value. Consequently, a mixed solution comprising a non-fluorescent glucose solution and sodium humate solution is formulated as the COD testing solution for the Laser-Induced Fluorescence imaging method. Based on prior research [28], it has been demonstrated that by modifying the ratio of sodium humate to glucose, the fluorescence intensity of a test solution can be aligned with that of natural water samples. When the COD value, ascertained through the rapid digestion spectrophotometric method, matches the integrated fluorescence intensity derived from spectroscopy, it can be inferred that the test solution and natural water samples have achieved the optimal ratio. The procedure to ascertain this optimal ratio involves several steps: initially, the COD value of natural water samples collected from Riyue Lake at the Harbin Institute of Technology (Weihai) is measured using rapid digestion spectrophotometry. Subsequently, mixed solutions with varying ratios are prepared under identical COD conditions as with natural water. Finally, adjustments are made iteratively until the spectral integral fluorescence intensity of the natural water aligns with that of the test solution. The optimal ratio determined through this method is 1/29.

The water quality detection system, as illustrated in Figure 2, was employed to analyze laboratory samples with varying concentrations. Fluorescence signals were acquired and processed through image analysis. During the experiment, COD testing solutions were prepared with concentrations ranging from 0 to 18 mg/L in increments of 1 mg/L. The laser intensity was modulated according to experimental requirements, revealing a proportional relationship between laser intensity and fluorescence signal. This strong correlation indicates that excessive laser intensity results in saturation of the optical image, while insufficient intensity leads to weak fluorescence signals. Consequently, to extend the detection range for organic matter concentration, the laser power of the system was optimized based on a series of preliminary experiments and theoretical calculations. Ultimately, the laser power was set at 45 mW.

**Figure 4:**
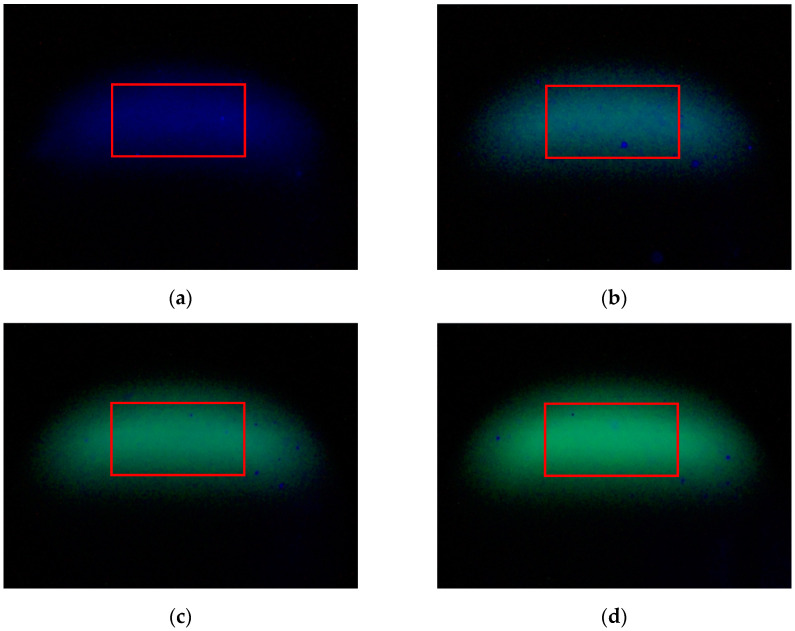
Fluorescence images of testing solutions with different concentrations collected by the system. The red rectangular frame indicates the Region of Interest (ROI) selected for analysis. (**a**) 0 mg/L COD testing solution; (**b**) 4 mg/L COD testing solution; (**c**) 8 mg/L COD testing solution; (**d**) 12 mg/L COD testing solution.

Figure 4 presents the fluorescence images of COD testing solutions at varying concentrations, obtained using a water quality detection system that employs LIF image processing. In Figure 4a, the image captured by the CMOS image sensor in the vertical orientation when the laser is directed at pure water, with blue being the dominant color. Figure 4b displays the fluorescence image of the COD testing solution at a concentration of 4 mg/L, characterized by a blue–green appearance. As the concentration of the testing solution increases to 8 mg/L, the image in Figure 4c reveals that the intensity of green surpasses that of blue. In Figure 4d, at a solution concentration of 12 mg/L, the green intensity markedly exceeds that of the other colors, resulting in a pronounced green coloration in the image.

In this study, a Region Of Interest (ROI) with a size of 240 × 130 pixels was selected within the effective area of the original image, as indicated by the red rectangular frame in Figure 4. This selection of ROI allows for more effective detection of fluorescence signals, reduces background noise interference, and improves the speed of image processing. During the image processing phase, noise generated by bubbles and suspended particles substantially influences the values of the blue channel. To mitigate this effect, a method for setting a brightness threshold specifically for the blue channel is implemented. By examining the distribution of pixel values in the blue channel within the ROI, an appropriate threshold is established. Pixels that deviate from the normal range, exceeding this threshold, are identified as potential noise sources and subsequently removed to minimize noise interference in further analyses. Additionally, to enhance data stability, multi-frame image averaging is utilized. This approach not only significantly improves the signal-to-noise ratio but also ensures the precise extraction of key data from the fluorescence signal. In the experimental setup, 50 images were collected for each sample test.

**Figure 5:**
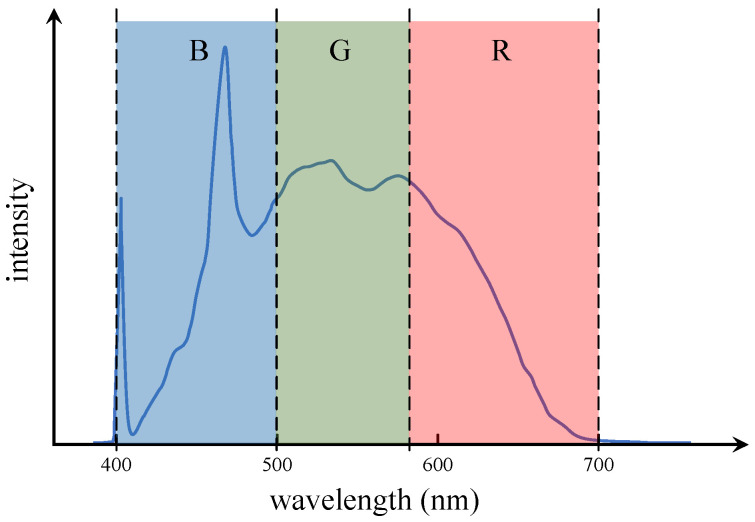
Correspondence between characteristic peaks in LIF spectrum of water and RGB spectral response of CMOS image sensor.

Upon excitation at a wavelength of 405 nm, the fluorescence spectrum of the water sample exhibits three prominent characteristic peaks: the laser peak at 405 nm, the water Raman peak at 471 nm, and the organic fluorescence peak near 530 nm [29]. The CMOS image sensor employed in this system adheres to the Bayer pattern, and each RGB channel has distinct spectral ranges: the blue channel (B) mainly spans approximately 400–500 nm, the green channel (G) mainly covers approximately 500–580 nm, and the red channel (R) mainly encompasses approximately 580–700 nm. As illustrated in Figure 5, there is a correlation between the positions of each characteristic peak in the LIF spectrum of water and the distinct color bands of the RGB channels. However, there exists an inherent overlap in the spectral response ranges of the Bayer pattern. The 405 nm peak is mainly detected by the blue channel (B), while the 471 nm peak is primarily captured in the blue–green overlap region, with the blue channel (B) contributing the dominant signal. The 530 nm peak is predominantly detected by the green channel (G), with a minor contribution from the red channel (R) in its lower-intensity region. During data acquisition, full-band spectral data, including overlapping regions between channels, were collected. Based on these spectral response characteristics, the water Raman scattering signal is predominantly detected through the blue channel (B), the high-intensity region of the organic fluorescence signal is primarily captured by the green channel (G), while its lower intensity region is detected by the red channel (R).

**Figure 6:**
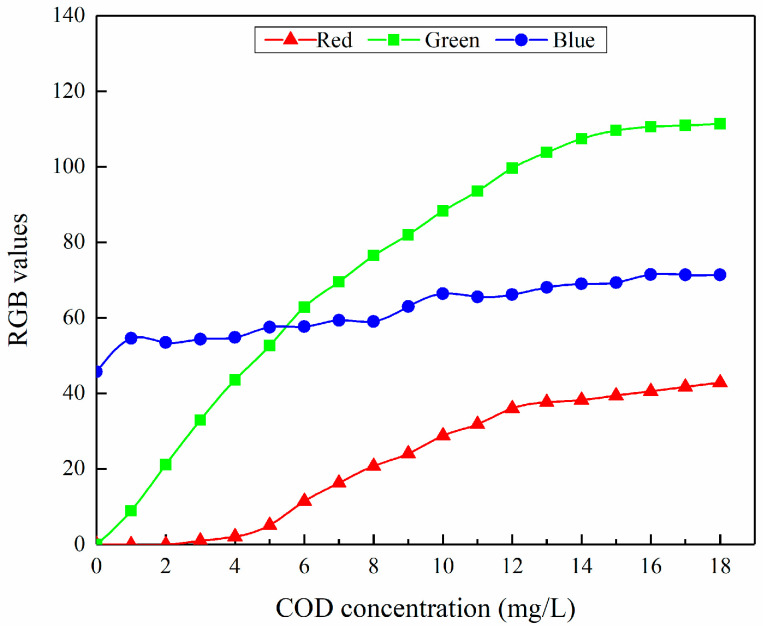
Variations in the RGB averages of the fluorescence image regions for COD samples at different concentrations.

The STM32 microcontroller is employed to separate the RGB color channels of the ROI from the collected fluorescence images and compute the regional average color value for each channel. To ensure system accuracy, 50 fluorescence images are collected for each COD concentration sample. The regional average values for R, G, and B of each image are calculated individually, followed by the computation of the overall average values from the 50 average values for each channel. These overall averages serve as the test data for the respective concentration. Figure 6 illustrates the test data and the variation trends of the color characteristic values R, G, and B of the fluorescence image regions extracted by the system. In the figure, the blue values predominantly arise from the scattering signals produced when the 405 nm laser has irradiated on water. Following filtration through a long-pass filter, the Rayleigh and Mie scattering signals from water molecules are substantially reduced. Consequently, the Raman scattering of water exerts a significant influence on the blue values. As the concentration of the testing solution increases, the scattering signals can be considered approximately constant, resulting in a relatively minor increase in the blue values, as depicted in Figure 6. The substantial increase in the green values with the concentration of the testing solution is attributed to the high fluorescence efficiency of organic matter, such as humic substances, in water at the green light band under 405 nm laser excitation. Conversely, the fluorescence efficiency of organic matter in the red light band is comparatively low, leading to a relatively modest increase in the red values with the concentration of the testing solution, as illustrated in Figure 6.

Furthermore, it can be seen from Figure 6 that when the COD sample concentration is in the range of 0–12 mg/L, the RGB values show a good linear correlation with the COD concentration of the testing solution. However, when the COD sample concentration exceeds 12 mg/L, the change in RGB values flattens significantly, tends to be stable, and presents a saturation phenomenon. Therefore, this study only selects the linear data within the range of COD concentration from 0 to 12 mg/L for subsequent analysis and excludes the saturated segment data above 12 mg/L. In this way, the interference of nonlinear errors is effectively avoided, and the accuracy and reliability of the system in detecting the COD concentration are improved.

According to the original data in the range of 0–12 mg/L in Figure 6, the obtained coefficients are shown in Table 1. The results indicate that there is a multiple correlation among the RGB values of the fluorescence image color parameters. There are also relatively strong linear correlation relationships between R, G, B, and the COD concentration values, among which the correlation between G and the COD concentration is the strongest.

Given the multiple correlations among RGB variables, this study employs Partial Least Squares Regression (PLSR) with a single dependent variable for multivariate statistical analysis. PLSR is a robust technique that effectively extracts information from multiple correlated independent variables and constructs an accurate predictive model for COD concentration values. Additionally, PLSR incorporates a dimensionality reduction mechanism, projecting the original high-dimensional data into a lower-dimensional space by identifying latent variables that capture the maximum covariance between the independent and dependent variables. This process not only reduces the complexity of the model but also mitigates noise and multicollinearity in the data, thereby enhancing the stability and interpretability of the predictive model [38].

Consequently, the regression equation for COD concentration, derived using the Partial Least Squares method with a single dependent variable, is as follows:

*C_COD_* = −2.1562 + 0.1013 × R + 0.0709 × G + 0.0479 × B (2)

Equation (2) represents the PLSR regression model based on the RGB image features. In the original training data, the model’s coefficient of determination R^2^ = 0.9975, and the Root Mean Square Error (RMSE) is 0.1865.

Following the construction of the model, a 10-fold cross-validation procedure was employed to rigorously assess its robustness and efficacy. The dataset was randomly partitioned into 10 distinct, non-overlapping subsets. In each iteration of the cross-validation, one subset was designated as the test set, while the remaining nine subsets were utilized for training purposes. This iterative process was conducted 10 times, resulting in 10 separate performance evaluations. Upon completion of the 10-fold cross-validation, the average coefficient of determination (R^2^) was found to be 0.996, which is marginally lower than the initial value of 0.9975 yet still indicative of a high degree of model fit. The average Root Mean Square Error (RMSE) was calculated to be 0.195, slightly exceeding the original value of 0.1865 but remaining within an acceptable range. These findings underscore the model’s strong predictive accuracy and generalizability across diverse data partitions, thereby effectively mitigating the risk of overfitting.

To intuitively assess the reliability of the regression equation, Table 2 presents the following information for each working point: 1. The original concentration values *C_COD_*; 2. The fitted values *C_m_* obtained from the regression model; 3. The differences *f_i_* between *C_COD_* and *C_m_*; 4. The relative errors *δ* between the model predictions and the actual values.

**Figure 7:**
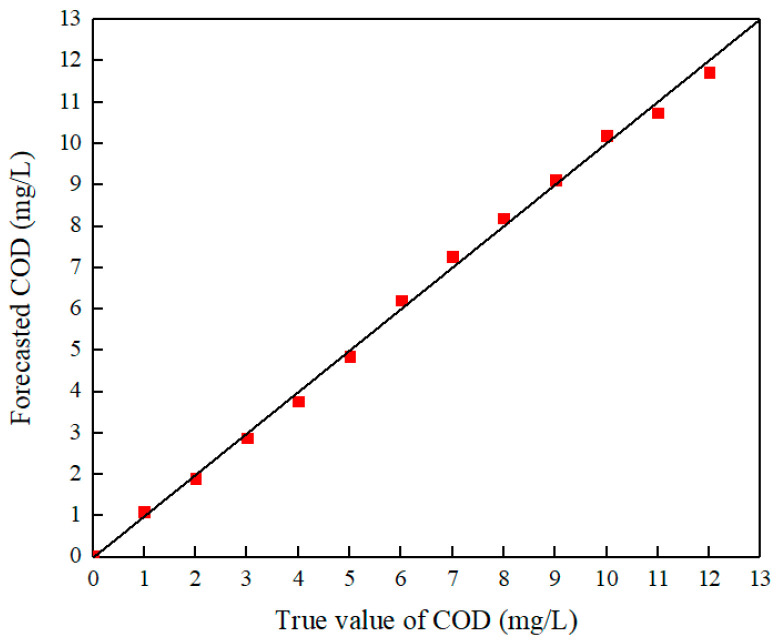
Relationship between the COD concentration values of the standard solution and the predicted values of the PLSR model based on the RGB of fluorescence images.

The results demonstrated that within the concentration range of 0–12 mg/L, the relative error of the prediction model remains below 10%. Meanwhile, the actual values of COD were compared with the predicted values, as shown in Figure 7. Both Table 2 and Figure 7 jointly indicate that the Partial Least Squares Regression model constructed by the system has a relatively high accuracy in predicting low concentrations of COD.

**Figure 8:**
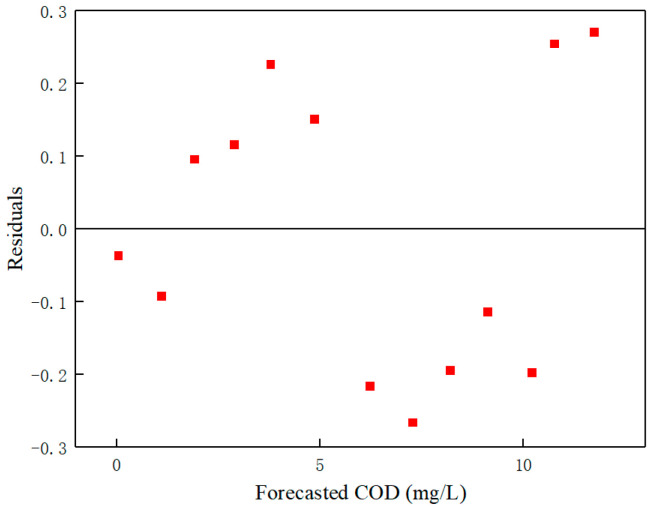
PLSR model residual plot.

**Figure 9:**
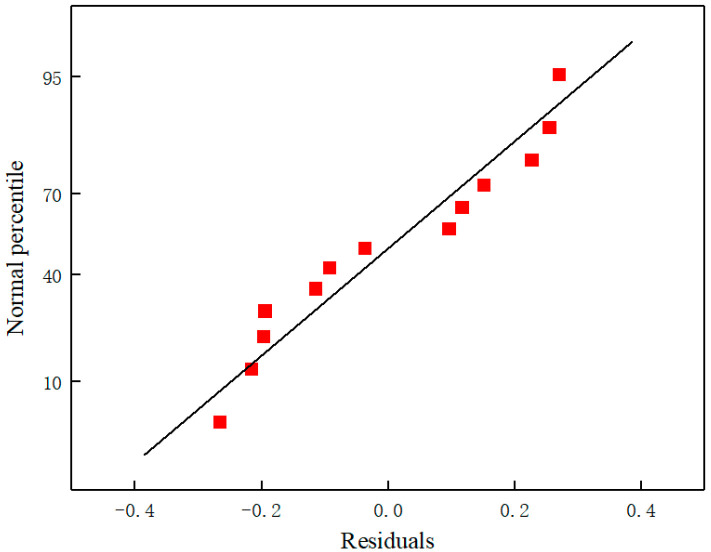
Normal probability plot of the residuals.

To further assess the applicability and limitations of the PLSR model, a comprehensive residual analysis was performed. Figure 8 illustrates the residual plot, where the residuals display a random distribution around zero without any discernible patterns or systematic trends. This observation suggests that the model assumptions are satisfied, indicating that the PLSR model is well-suited for the dataset. Notably, the majority of the residuals lie within the range of ±0.3 mg/L, which demonstrates the model’s high precision in predicting COD concentrations. Furthermore, the normal probability plot of the residuals (Figure 9) was utilized to evaluate the normality of the residuals. The plot shows that the residuals closely align with a straight line, confirming their normal distribution. This finding further substantiates the robustness of the PLSR model and its suitability for predicting COD concentrations based on RGB color features.

In summary, the results of the cross-validation and residual analysis collectively demonstrate that the PLSR model is both robust and reliable, capable of accurately predicting COD concentrations within the range of 0–12 mg/L. The model’s high coefficient of determination (R^2^ = 0.9974), low Root Mean Square Error (RMSE = 0.1865), and the random, normally distributed residuals underscore its strong predictive performance. These findings highlight the model’s potential for practical applications in real-time water quality monitoring, particularly for low-concentration COD detection.

To verify the reliability of the new method proposed in this study, comparative experiments were conducted between the new method and traditional chemical detection methods for actual water samples. For this purpose, five water samples from the MuZhu River in Weihai City, Shandong Province, China, were selected for analysis. In accordance with the Chinese National Standard GB/T 11892-1989 “Determination of Permanganate Index in Water Quality” [39], the permanganate index concentrations of these five samples were measured. Simultaneously, under the same experimental conditions, we also tested these samples using the LIF image processing system. The results of the water quality measurements are presented in Table 3.

As illustrated in Table 3, the permanganate index values determined by the LIF image processing method exhibit a high degree of consistency with those obtained through chemical analysis, with relative errors consistently below 10%. The results indicate that the proposed LIF image processing method fulfills the stringent requirements for accurate measurement of the permanganate index in practical water samples, underscoring its potential for application in environmental monitoring.

## 4. Conclusions

This paper proposes a new method for detecting low-concentration COD in water based on Laser-Induced Fluorescence (LIF) image processing, and an LIF image processing COD detection system was developed. The system mainly includes a CMOS image sensor, STM32 microprocessor, laser emission module, and display module. In the experiment, a mixed solution of sodium humate and glucose in a ratio of 1:29 was used as the testing solution for COD. The system conducted detection and analysis on samples with COD concentrations ranging from 0 to 18 mg/L, selecting an appropriate laser intensity. The fluorescence image data undergo color channel separation and feature processing. The Partial Least Squares Regression analysis method with a single dependent variable was adopted, and a prediction model for the COD concentration value based on the average RGB color features of the characteristic regions of fluorescence images was constructed. When the COD concentration is within the range of 0–12 mg/L, the relative error of the detection model is kept below 10%, with a coefficient of determination of 0.9974 and a root square error of 0.1865, which meets the accuracy requirements for detection.

The primary contributions of this study are delineated as follows: (1) The study introduces an innovative detection method that intricately integrates multidisciplinary technologies, including optics, image processing, and data analysis, thereby offering a novel perspective for assessing water quality. (2) A rapid, real-time, and non-contact water quality detection system has been developed, which markedly enhances the timeliness, adaptability, and efficiency of the detection process, rendering it highly suitable for practical environmental monitoring applications. (3) The system exhibits high sensitivity and robust stability in detecting low-concentration organic compounds in water, thereby providing reliable technical support for early warning of environmental pollution and facilitating water resource management and environmental protection. (4) The system features notable advantages, such as a simple structure, low cost, and rapid detection capabilities. It not only enables real-time monitoring but can also be seamlessly integrated with cloud systems for remote data analysis and storage, thereby broadening its applicability in diverse and complex real-world scenarios and significantly enhancing its practical value.

Nonetheless, this system is constrained by certain limitations. Firstly, there is an intrinsic spectral overlap among the RGB channels of the CMOS sensor, resulting in color crosstalk during the spectral-to-RGB conversion process. While this approach simplifies the experimental setup and data processing, thereby enhancing its applicability across various CMOS sensors and fluorescence spectra, it does so at the expense of high-resolution spectral mapping. Consequently, future research should be dedicated to exploring more precise mapping techniques and calibrating the spectral response sensitivity in the overlapping regions of the RGB channels, aiming to reduce color crosstalk and enhance accuracy. Secondly, the fluorescence effects of different organic compounds in complex water bodies vary significantly, leading to overlapping characteristic peaks and potential interference, which limits the system’s ability to accurately identify specific organic compounds. Therefore, extensive experimental analyses are required in the future to establish a comprehensive fluorescence spectral database for various organic compounds. Thirdly, the current RGB-based analysis system exhibits limited spectral resolution, which impairs the precision of organic matter detection. To address this issue, future investigations could consider the integration of hyperspectral or multispectral cameras. Such integration would facilitate higher spectral resolution, thereby improving the reliability of water quality monitoring and early-warning capabilities.

In conclusion, the COD detection system based on the LIF image processing method designed in this paper is suitable for the detection of organic matter at relatively low concentrations in water. It has the advantages of high sensitivity, strong stability, non-contact, and rapid detection speed, showing great application potential in rapid water quality monitoring and early warning. This method provides a novel technical approach to the field of water quality monitoring and holds significant importance for environmental protection and water resource management.

## Figures and Tables

**Table 1:** Correlation coefficient matrix.

	G	B	*C_COD_*/(mg/L)
R	0.9333	0.8994	0.9707
G		0.9354	0.9899
B			0.9440

**Table 2:** Comparison of fitted values.

*C_COD_*/(mg/L)	*C_m_*/(mg/L)	*f_i_*/(mg/L)	*δ*/(%)
0	0.036	−0.036	
1	1.092	−0.092	9.232
2	1.904	0.096	4.802
3	2.883	0.117	3.886
4	3.773	0.227	5.670
5	4.849	0.151	3.024
6	6.216	−0.216	3.599
7	7.266	−0.266	3.799
8	8.195	−0.195	2.432
9	9.114	−0.114	1.267
10	10.197	−0.197	1.972
11	10.745	0.255	2.318
12	11.729	0.271	2.256

**Table 3:** The results of water quality measurement.

Sample Number	Permanganate Index/(mg/L)		Relative Error (%)
	Chemical Analysis	LIF Image Processing	
1	2.825	3.034	7.398
2	4.532	4.086	9.841
3	4.680	4.489	4.076
4	5.800	5.727	1.256
5	6.135	5.976	2.655

## Data Availability

Data are contained within the article.

## Abbreviations

The following abbreviations are used in this manuscript:

COD	Chemical Oxygen Demand
PLS	Partial Least Squares Regression
UV-Vis	Ultraviolet–Visible Spectroscopy
BOD	Biochemical Oxygen Demand
DOM	Dissolved Organic Matter
PCA	Principal Component Analysis
CCD	Charge-Coupled Device
OECD	Organization for Economic Cooperation and Development
LIF	Laser-Induced Fluorescence
CMOS	Complementary Metal Oxide Semiconductor
MCU	Microcontroller Unit
DCMI	Digital Camera Interface
ROI	Region Of Interest
